# Developing a simple artificial intelligence fuzzy-based model for estimating saturated hydraulic conductivity of soil

**DOI:** 10.1038/s41598-025-13029-9

**Published:** 2025-08-04

**Authors:** Mohammad Naderianfar

**Affiliations:** Water Science and Engineering Department, University of Jiroft, Jiroft, Iran

**Keywords:** UNSODA, Soil texture, Fuzzy inference system, Linear regression, Hydrogeology, Engineering

## Abstract

Saturated hydraulic conductivity is one of the important physical properties of soil in modeling water and solute transport, irrigation management, and drainage issues. Laboratory and field methods for directly measuring this parameter are time-consuming and costly. In recent years, the use of intelligent systems for estimating various soil parameters has significantly increased. Therefore, this research aims to utilize Fuzzy Inference Systems (FIS), Artificial Neural Networks (ANN), and Linear Regression (LR) to create a mapping between soil texture parameters and saturated hydraulic conductivity. The data used in this study includes physical properties related to 331 soil samples from the UNSODA soil database (170 samples) and existing data from soils in the cities of Amol, Babol, Karaj (50 samples), and Shahrekord (111 samples). After examining different models and combinations of available data, three models were proposed for estimating saturated hydraulic conductivity. In these models, saturated hydraulic conductivity was estimated using soil texture characteristics (percentage of clay, silt, and sand) and bulk density, and the performance of the models was evaluated using statistics such as root mean square error (RMSE), mean bias error (MBE), and coefficient of determination (R^2^). Comparing the results of the proposed nonlinear models with different input parameters showed that fuzzy systems can estimate saturated hydraulic conductivity with acceptable accuracy. During the training phase, the fuzzy model with four input variables (percentage of clay, silt, sand, and bulk density) had the highest correlation (r = 0.92), and considering other evaluation parameters (R^2^ = 0.84, MBE = 0.28 cm/hr, and RMSE = 1.64 cm/hr), it showed a good fit with the measured values. In the testing phase, similar results were obtained, and the fuzzy model with four parameters had the best fit. Based on the results of this research, the fuzzy model can be introduced as one of the methods for estimating saturated hydraulic conductivity with suitable accuracy.

## Introduction

Quantitative expression of the hydraulic properties of soil is essential in many studies related to flow in porous media that use numerical models to simulate the movement of water and solutes. Direct measurement of these properties, whether in the field or laboratory, is very time-consuming, costly, and challenging^1^. Additionally, due to the temporal and spatial variability of soil properties, these measurements may not represent the actual characteristics of the soil unless a large number of samples are collected. Therefore, indirect methods have been proposed as a suitable approach to relatively address these issues^2,3^). One of the indirect methods for estimating saturated hydraulic conductivity is the use of transfer functions. Transfer functions estimate the hydraulic properties of soil based on readily available physical characteristics such as texture, bulk density, and organic matter content^4,5^. Saturated hydraulic conductivity is generally influenced by two factors: the hydraulic properties of the fluid and the characteristics of the porous medium of the soil. Porous medium characteristics include total porosity, shape and geometry of pores, and pore size distribution. Therefore, various factors such as texture, structure, organic matter content, and salinity concentration that affect the characteristics of the porous medium can influence saturated hydraulic conductivity. Because saturated hydraulic conductivity depends on these factors, using mathematical models considering all influencing factors is challenging and often associated with significant errors. Additionally, the uncertainty and inaccuracy in estimating saturated hydraulic conductivity play a major role in the complexity of this phenomenon, as this uncertainty depends on soil conditions and its properties, while inaccuracy relates to human or measurement errors^6^.

In recent years, the use of artificial intelligence models for simulating various issues has become more prevalent^7^. Artificial neural network models and fuzzy inference systems are two prominent examples of these models. The most important aspect of modeling artificial neural networks is selecting appropriate inputs to achieve the desired output. Alongside this, the structure of the artificial neural network and how to choose between neurons and the weights assigned to each neuron are also important^8^. Numerous studies have been conducted on modeling soil hydraulic conductivity using linear and nonlinear regression methods^8^. However, due to the ability of nonlinear models, including artificial neural networks and fuzzy logic, to model complex processes with numerous influencing factors, their application in soil science has expanded^9^. For instance, Trejo-Alonso et al.^7^ employed artificial neural networks to estimate saturated hydraulic conductivity. The results show that our artificial neural networks obtained from 0.0459 to 0.0413 in the RMSE measurement, and 0.9725 to 0.9780 for R^2^, which are in good agreement with other works. They also showed that reducing the amount of the input data offered us better results. Faloye et al.^10^ utilized multiple linear regression (MLR), artificial neural networks (ANN), and adaptive neuro-fuzzy inference systems (ANFIS) in a study to find the relationship between soil moisture content and saturated hydraulic conductivity in biochar-amended soil. More et al.^11^ applied ELM, SVM, and ANFIS models for modeling saturated hydraulic conductivity in semi-arid tropical soils in India. Their results indicated that in the Punanaka and Murarji Peth areas, the ELM model had higher accuracy, while in the Mulegoan area, ANFIS models performed better (r = 0.8). Sihag et al.^3^used artificial neural networks and fuzzy models to predict saturated hydraulic conductivity. The main goal of their research was to develop models based on fuzzy logic and neural networks to estimate Ks. Their comparison results showed that using artificial neural networks with a correlation coefficient of 0.866 could effectively predict Ks (RMSE = 4.56 cm/h). Norouzian et al^12^. evaluated regression models and artificial neural networks in estimating saturated hydraulic conductivity in Mazandaran. To model saturated hydraulic conductivity, they employed multiple linear regression (MLR), multilayer perceptron (MLP), and radial basis function (RBF) neural networks. Their results indicated that using a multilayer perceptron neural network with two hidden layers is an efficient method for determining saturated hydraulic conductivity in the region, which can estimate saturated hydraulic conductivity while saving time and costs. Hekmatzad et al.^13^ estimated saturated hydraulic conductivity in some soils of Ilam Province using artificial neural networks and regression methods. Their results showed that when only a few readily available soil features are accessible, regression transfer functions and artificial neural networks can predict Ks with relatively good accuracy. Overall, their results indicated that artificial neural networks have relatively better performance than linear regression models in estimating saturated hydraulic conductivity of soils.

Yazdani et al.^14^ estimated saturated hydraulic conductivity using regression methods and adaptive neuro-fuzzy inference systems through readily available parameters. In their studies, the best regression model for the transfer function was obtained with inputs including the percentage of sand, bulk density, real density, fractal dimension of particle size, porosity, and percentage of silt. For the adaptive neuro-fuzzy inference system, the inputs included bulk density, real density, porosity, and the fractal dimension of particle size, with a single output layer showing the best performance. Model evaluations indicated that these models are suitable for estimating saturated hydraulic conductivity in soils with light to medium texture (sandy loam, loam, and silt loam). Azadmard et al.^15^ investigated the application of a hybrid genetic algorithm-artificial neural network (GA-ANN) approach for predicting near-saturated soil hydraulic properties in the Moghan region. Their results demonstrated that the GA-ANN model significantly outperformed linear regression, explaining up to 80% of the variability in the data. These findings highlight the importance of utilizing intelligent hybrid models to enhance the accuracy of soil hydraulic property predictions. Ross et al.^16^ applied fuzzy inference systems to estimate hydraulic conductivity using soil particle size distribution data obtained from the Superfund site, concluding that it yielded better results compared to other methods^16^. Nadiri et al.^17^ used a Bayesian artificial intelligence model to estimate average hydraulic conductivity. In their research, they presented three models: fuzzy, neural network, and adaptive neuro-fuzzy, concluding that among the presented models, the results of the fuzzy and neural network models were similar, while the adaptive neuro-fuzzy model yielded weaker results^17^.

Although different input data were used for the models in the conducted studies, and the presented models were compared using various methods, almost all results of these investigations showed that these models are suitable tools for estimating hydraulic conductivity and can be recommended for estimating this parameter. Considering the results of the above studies regarding the capabilities of neural network and fuzzy logic models in estimating hydraulic conductivity, this research aims to investigate the feasibility of using expert systems to create a mapping between the parameters of soil texture and saturated hydraulic conductivity. Furthermore, due to incomplete information on one hand and the high cost of recording data for soil parameters on the other, an attempt has been made to provide a model using the minimum necessary data.

## Materials and methods

**Fig. 1:**
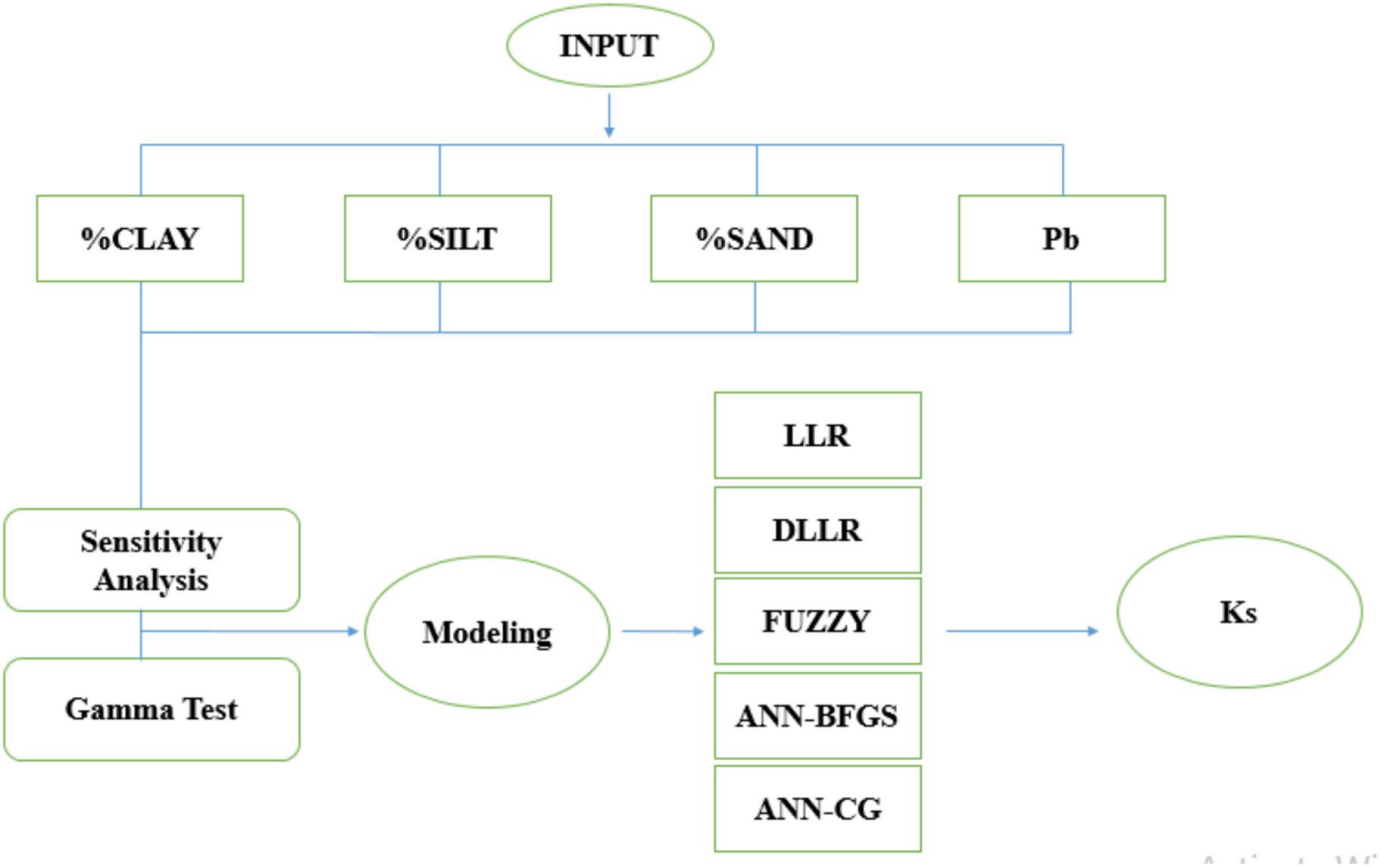
Flowchart for the modelling of saturated hydraulic conductivity of soil.

In this study, 331 soil samples, comprising 10 different texture classes, were utilized from the UNSODA soil database (170 samples) and existing data from the soils of the cities of Amol, Babol, Karaj (50 samples), and Shahrekord (111 samples). Table 1 presents the average information and specifications of some of the soils used. The input parameters for modelling the saturated hydraulic conductivity of soil include the percentages of clay, silt, and sand, and the bulk density (Fig. 1).

**Table 1:** Specifications of the soils used.

Soil texture	Clay (%)	Loam (%)	Sand (%)	*p*_b_	Ks
Clay	48.40	33.20	18.42	1.39	0.45
Loam	23.26	33.21	43.60	1.46	3.17
Sandy loam	16.40	28.60	55	1.42	5.60
Clay loam	32.40	33.9	33.60	1.55	1.93
Silt loam	18.42	51	30.62	1.43	0.88

### Gamma test

The gamma test is a nonlinear modeling tool that allows for the examination of suitable combinations of input parameters for modeling output data and creating a smooth model. The gamma test was first reported by^18^) and has since been discussed in detail by many researchers such as^19^)^20^,), and Evans and Jones^21^. When the factors influencing a phenomenon are significantly numerous, the gamma test can determine the order of importance of input variables and the best combination among all possible combinations. By accepting an error value in obtaining the output from the input value and due to the complexity and nonlinearity of the modeled phenomena, the gamma test expresses this error as the following relationship between the input and output datasets with function (f):

1 $$y_{i} = f(x_{1} ,x_{2} ,...,x_{m} ) + r$$

where xi are the input data, y is the output, f represents the smooth function used for modeling the data, and r denotes the random variable used to represent the error. The gamma statistic is equal to the intercept of the regression line created; whose equation is as follows:

2 $$\gamma = A\delta + \Gamma$$

The statistic Γ is the variance of the model’s output, which cannot be calculated with a simple model and traditional methods. Additionally, A calculating the slope of the regression line can provide useful information regarding the complexity of the system under study.

For the results of the gamma test to be meaningful, two main prerequisites must be met. First, the sampling of the input data vector must be such that as the number of data points increases, the distance between x(i) and the nearest neighbor x(j) approaches zero. Second, this model assumes that the noise distribution r in each output y is independent of the input x. Specifically, the noise variance for a given output is constant. The gamma test returns an estimate of the mean noise variance and can provide useful information when selecting relevant inputs^19^. The gamma test is a non-parametric method, and regardless of the specific techniques used to construct the subsequent model, the results are obtained. Therefore, by considering another term (V-Ratio), which indicates a fixed noise estimate on a scale from zero to one, the result can be standardized. V-Ratio is defined as follows:

3 $$V_{ratioo} = \frac{\Gamma }{{\delta^{2} (y)}}$$

where $\delta^{2} (y)$ is the variance of the output y, allowing for an independent judgment about how the output is modeled by a smooth function. A V-Ratio close to zero indicates a high degree of predictability of the output by the input data.

In this research, to calculate the gamma test statistic, the Win Gamma software was used for preprocessing, followed by determining the relationship between the variables and the output. A low standard error indicates a better fit of the model, and gamma is equivalent to the variance of the model error constructed with these data. The V-Ratio index also indicates the extent of output modeled by the function, and the closer it is to one, the less well the model can estimate the output, or in other words, the output is equivalent to random noise. Therefore, the closer the gamma statistic and the V-Ratio index are to zero, the more accurately the output can be estimated using these data, making them suitable for modeling.

## Nonlinear models

### Fuzzy model structure

Fuzzy inference systems are nonlinear models that describe the input–output relationship of a real system using fuzzy “if–then” rules. The general structure of the formulated rules is as follows:

4 $${\text{Rule m }}:{\text{ IF }}\left( {{\text{X1 is A1}},{\text{ m}}} \right) {\text{AND }}\left( {{\text{X2 is A2}},{\text{ m}}} \right) {\text{AND }}\left( {{\text{Xk is Ak}},{\text{ m}}} \right){\text{ THEN Y is }} \ldots$$

In other words, a fuzzy rule expresses the relationship between k input variables X1, X2…. Xk and output Y. The term A_k,m_ in the antecedent part of the rules represents the fuzzy sets used to partition the input space into overlapping regions. A fuzzy set is a generalization of classical sets, where the membership function is defined as a matter of degree in binary form (either non-membership or full membership)^22^. To fuzzification the input and output indices, the range of their variations (based on available information) was first examined, and an appropriate number of levels was considered for the input and output variables of the model. In this study, for the parameters of clay percentage (CL), sand percentage (SA), silt percentage (SI), and bulk density (ρ), 39, 39, 39, and 18 intervals were used based on the available data, respectively. Additionally, the model’s output was also considered with 40 levels corresponding to input variations during the model training period. Furthermore, to determine the fuzzy membership functions of the input and output variables and the degree of overlap of the fuzzy functions, both the physical characteristics of the issue under discussion and expert opinions were utilized. Given the widespread application of triangular and trapezoidal membership functions in practical problems and the results obtained from the studies, both of these functions were used for fuzzifying the input and output variables in this research.

### Definition of fuzzy rules and function composition

Based on the membership functions of the input and output variables and their degree of overlap, as well as the relationship between input and output in the training phase of the model, various rules with different weights were defined. To complete the modeling stages, this research employed the Mamdani and Implication method for fuzzy inference, the minimum method for necessity, and the maximum method for aggregating fuzzy rules^23,24^.

In fuzzy modeling, the final inference leads to a fuzzy result; thus, to obtain a real number, various defuzzification methods proposed by researchers must be used. The most important of these methods include the maximum average method, the centroid method, the bisector intersection method, etc. In this research, considering the comprehensiveness of the centroid method, it was utilized^25^.

### Artificial Neural Network (ANN) model

Artificial neural networks, like natural neural networks, are composed of components called neurons. Just as in a natural neural network, some cells are responsible for receiving external stimulus effects, some process information, and some transmit the response to the target organ, in artificial neural networks, some cells receive the problem information, some process the data, and some provide the solution to the problem. There are two distinct phases in the use of neural networks that complement each other, ultimately leading to the use of the network. Initially, the network cells are trained, determining the appropriate weights. In the next phase, which is the main phase, the trained network is utilized. There are many training algorithms in neural networks, including the Back-Propagation Algorithm (BPA), the Gradient Descent Algorithm (CGA), the Momentum Algorithm (MA), and the Levenberg–Marquardt Algorithm (LMA). In this research, the BFGS learning algorithm and the Gradient Descent Algorithm (CGA) were used with the help of WinGamma software version 1.97^26–28^.

### Dynamic Local Linear Regression (DLLR)

The dynamic local linear regression method is widely used for examining parametric equations, studies related to short-term predictions, and smooth equations. In this method, the number of data points used in modeling, denoted as »pmax», meaning the penetration statistic, must be determined. Considering the neighboring points pmax, we need to solve a linear matrix equation:

5 $${\text{Xm}} = {\text{y}}$$

where X is the p_max_ × d matrix, with p_max_ input points represented as vectors, xi(1 < i < pmax) being the nearest neighboring points. y is the column vector length of p_max_ corresponding to the output, and m is the column vector of parameters that must be determined by providing an optimal mapping from x to y, such that:

6 $$\left( {\begin{array}{*{20}l} {x_{11} } \hfill & {x_{12} } \hfill & {x_{13} } \hfill & \ldots \hfill & {x_{1d} } \hfill \\ {x_{21} } \hfill & {x_{22} } \hfill & {x_{23} } \hfill & \ldots \hfill & {x_{2d} } \hfill \\ \vdots \hfill & \vdots \hfill & \vdots \hfill & \ddots \hfill & \vdots \hfill \\ {x_{{x_{p\max } 1}} } \hfill & {x_{{x_{p\max } 2}} } \hfill & {x_{{x_{p\max } 3}} } \hfill & \ldots \hfill & {x_{{x_{p\max } d}} } \hfill \\ \end{array} } \right)\left( {\begin{array}{*{20}l} {m_{1} } \hfill \\ {m_{2} } \hfill \\ {m_{3} } \hfill \\ \vdots \hfill \\ {m_{d} } \hfill \\ \end{array} } \right) = \left( {\begin{array}{*{20}l} {y_{1} } \hfill \\ {y_{1} } \hfill \\ \vdots \hfill \\ {y_{p\max } } \hfill \\ \end{array} } \right)$$

The rank r of matrix X is the number of linearly independent rows, which will be influenced by the existence or independent solution for m. If matrix X is square and independent, the independent solution of Eq. (10) will be m = x^−1^y. If X is not square or independent, we must try to find the vector m obtained by minimizing the following equation:

7 $$\left| {x_{m} - y} \right|^{2}$$

Thus, LLR algorithms can be used with the minimum number of direct evaluations.

### Training and test datasets for fuzzy models

For training and testing the model in this study, initially, existing data for saturated hydraulic conductivity from the UNSODA soil database and samples from various locations in the country were examined. This included 50 samples from Amol, Babol, and Karaj, along with 111 samples from Shahrekord that were available. Before analyzing the data, their quality was assessed; thus, some outlier data were empirically identified and removed from the dataset. Only data points with saturated hydraulic conductivity less than 20 cm per hour were considered. After eliminating outliers, a suitable total of 331 soil sample data points with various textures remained. With this number of data points, 80% of the data were used for training nonlinear models, and 20% were used for testing the models.

### Model evaluation

There are various criteria for evaluating predictive models, primarily based on the difference between predicted outputs and actual desired outputs. In this research, three parameters were used to evaluate experimental methods and fuzzy models: Root Mean Square Error (RMSE), Mean Bias Error (MBE), and the Coefficient of Determination (R^2^).

8 $$RMSE = \sqrt {\frac{1}{N}\sum\limits_{i = 1}^{N} {(P_{i} - O_{i} )^{2} } }$$

9 $$MBE = \frac{1}{N}\sum\limits_{i = 1}^{N} {(P_{i} - O_{i} )^{{}} }$$

10 $$R^{2} = \frac{{\left[ {\sum\limits_{i = 1}^{N} {\left( {P_{i} - \overline{P} } \right)\quad \left( {O_{i} - \overline{O} } \right)} } \right]^{2} }}{{\sum\limits_{i = 1}^{N} {\left( {P_{i} - \overline{P} } \right)^{2} \quad \sum\limits_{i = 1}^{N} {\left( {O_{i} - \overline{O} } \right)^{2} } } }}$$

where:

N is the number of samples, P_i_ are the values predicted by the model, O_i_ are the actual values, $\overline{P}$ is the mean of the predicted values, $\overline{O}$ is the mean of the actual values.

## Results and discussion

### Sensitivity analysis of input parameters

The percentages of clay, silt, sand, and bulk density (P_b_) were used as input variables for developing linear and non-linear models to predict saturated hydraulic conductivity. Table 2 summarizes the evaluation of different input combinations. The model incorporating all four variables (Scenario 1) demonstrated superior performance, with the lowest standard error (0.014), V-ratio (0.651), and Gamma value (0.162), reflecting high accuracy and model stability. Excluding bulk density resulted in decreased model performance; for example, the texture-only model (Scenario 6) showed increased standard error (0.022) and Gamma (0.195). Single-variable models, such as Scenario 8 (clay only), further exhibited reduced accuracy and higher sensitivity (Gamma = 0.236; standard error = 0.021). These findings underscore the essential contribution of bulk density to model robustness and predictive reliability. The Gamma coefficient proved to be an effective metric for assessing model sensitivity, with higher values correlating with increased prediction errors and reduced reliability. Based on the lowest Gamma coefficients and standard error values, Scenarios 1, 2, and 3 were selected for model development. Scenario 1, which incorporated all four input variables, demonstrated the highest predictive accuracy. Scenarios 2 and 3, utilizing three and two inputs respectively, also provided satisfactory performance. This selection is expected to play a significant role in improving the stability and enhancing the predictive reliability of both linear and non-linear models for estimating saturated hydraulic conductivity.

**Table 2:** Quantitative sensitivity analysis of soil texture and bulk density inputs on Ks estimation.

Scenario	Input combination	Variables	Gamma	Gradient	Standard error	V-Ratio
1	1111	Clay, Silt, Sand, P_b_	0.162	0.148	0.014	0.651
2	1011	Clay, Sand, P_b_	0.156	0.309	0.016	0.626
3	0011	Sand, P_b_	0.166	0.086	0.018	0.667
4	0001	P_b_	0.228	−0.398	0.013	0.914
5	1001	Clay, P_b_	0.192	−0.060	0.021	0.770
6	1110	Clay, Silt, Sand	0.195	0.197	0.022	0.781
7	1100	Clay, Silt	0.195	−0.014	0.030	0.781
8	1000	Clay	0.236	−5.251	0.021	0.945
9	0100	Silt	0.216	6.789	0.030	0.865
10	0010	Sand	0.208	18.198	0.020	0.835
11	0110	Silt, Sand	0.211	−0.626	0.036	0.844
12	0101	Silt, P_b_	0.204	−0.086	0.017	0.817
13	1010	Clay, Sand	0.176	1.324	0.025	0.705
14	0111	Silt, Sand, P_b_	0.174	0.098	0.022	0.699
15	1101	Clay, Silt, P_b_	0.183	0.160	0.012	0.732

### Fuzzy models

As mentioned, to create Fuzzy models in this study, the measured values of saturated hydraulic conductivity were used as outputs in the models, and different combinations of parameters affecting saturated hydraulic conductivity (Table 2) were considered as model inputs. To assess the performance of the Fuzzy models, evaluation criteria (correlation coefficient, mean bias error, root mean square error) were used. The results showed a high correlation between the proposed fuzzy models and the measured values of saturated hydraulic conductivity (Table 3). The highest correlation among the fuzzy models was found for the four-parameter model (percent clay, percent sand, percent silt, and bulk density), while the lowest was for the two-input model with percent sand and bulk density. These results indicate that the fuzzy model can accurately compute saturated hydraulic conductivity with various input variables. By calculating other performance evaluation parameters for the fuzzy models presented during the training phase, the Mean Bias Error (MBE) ranged from 0.280 to 0.950 cm per hour, indicating a high accuracy of the developed fuzzy models. Furthermore, based on the results for MBE, it can be stated that the values predicted by the fuzzy models with different inputs were generally higher than the measured values, suggesting that the models tended to overestimate.

**Table 3:** Evaluation results and performance criteria for different models predicting saturated hydraulic conductivity during the training and testing phases.

Scenario	Model	Input	Train			Test		
			R^2^	RMSE	MBE	R^2^	RMSE	MBE
1	DLLR	1111	0.990	0.006	0.0037	0.341	0.428	− 0.159
2		1011	0.992	0.004	0.0003	0.402	0.402	− 0.141
3		0011	0.984	0.006	0.0003	0.391	0.418	0.144
1	ANN_BFGS_	1111	0.297	0.420	0.0003	0.213	0.434	− 0.100
2		1011	0.293	0.422	0.0110	0.276	0.407	− 0.060
3		0011	0.156	0.431	0.0003	0.205	0.422	0.031
1	ANN_C-G_	1111	0.296	0.420	0.0004	0.188	0.431	− 0.062
2		1011	0.295	0.422	−0.0040	0.293	0.411	− 0.091
3		0011	0.183	0.453	0.0004	0.125	0.434	0.024
1	FUZZY	1111	0.841	1.640	0.2800	0.832	1.650	0.360
2		1011	0.810	1.820	0.3800	0.617	2.620	0.831
3		0011	0.556	2.990	0.9500	0.392	3.420	1.320
1	Linear regression	1111	0.214	3.488	0.0001	0.301	3.319	0.430
2		1011	0.213	3.488	0.0001	0.302	3.299	− 0.140
3		0011	0.139	3.650	0.0001	0.189	3.532	0.177

**Fig. 2:**
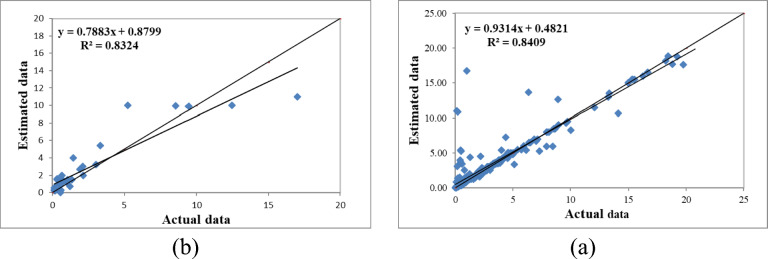
Comparison of the fuzzy model’s calculated saturated hydraulic conductivity with four inputs (**a**—model training) and (**b**—model testing).

Examining and comparing the RMSE values presented in Table 3 also indicates that the individual predicted values in the fuzzy model correspond closely to the measured values. The statistical variable for the presented fuzzy models varied between 1.640 to 2.990 cm per hour, indicating the adequate precision of the fuzzy inference system. Additionally, the four-parameter model with inputs of percent sand, percent clay, percent silt, and bulk density performed similarly to the three-parameter model with percent clay, sand, and bulk density. The results from these two models suggest that the percent silt parameter had little impact on estimating saturated hydraulic conductivity in the examined samples. Moreover, in the two-parameter model, by removing the percent clay parameter, it was observed that the model’s performance significantly decreased, leading to the conclusion that the percent clay in the soil is an influential parameter for estimating saturated hydraulic conductivity. This finding was not surprising given the unique characteristics of clay particles. To determine the best model, rankings based on various criteria (R^2^, RMSE, MBE) were used, and ultimately, the model with the lowest total rank was selected as the best model. The ranking results during the training phase indicated that the four-variable model with inputs of percent clay, sand, silt, and bulk density performed better (Table 3 and Fig. 2a).

Figure 2 presents the results of the fuzzy modeling of saturated hydraulic conductivity with four inputs (clay, sand, and silt percentages, and bulk density) during the model training and testing phases. The evaluation metrics R^2^, RMSE, and MBE yielded values of 0.841, 1.640, and 0.280 during the training phase and 0.832, 1.650, and 0.360 during the testing phase, respectively. Ranking results based on the combined criteria also indicated that the fuzzy model with four inputs exhibited higher accuracy compared to the other models.

**Fig. 3:**
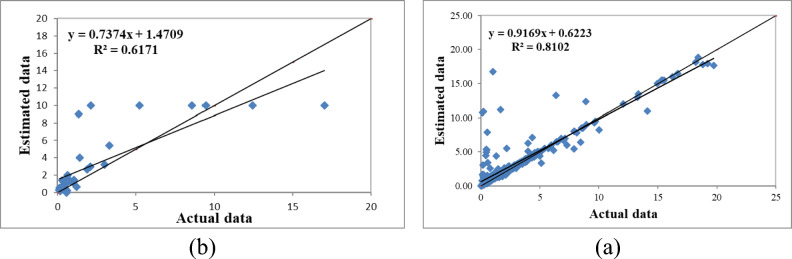
Comparison of the fuzzy model’s calculated saturated hydraulic conductivity with three inputs (**a**—model training) and (**b**—model testing).

Figure 3 presents the results of the fuzzy modeling of saturated hydraulic conductivity with three inputs (clay and sand percentages, and bulk density) during the model training and testing phases. The evaluation metrics R^2^, RMSE, and MBE yielded values of 0.810, 1.820, and 0.380 during the training phase and 0.617, 2.620, and 0.831 during the testing phase, respectively.

**Fig. 4:**
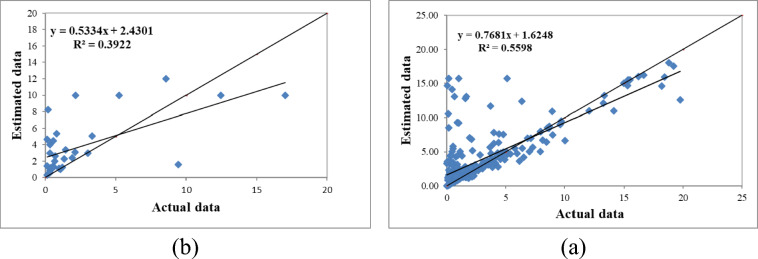
Comparison of the fuzzy model’s calculated saturated hydraulic conductivity with two inputs (a- model training) and (b- model testing).

Figure 4 presents the results of the fuzzy modeling of saturated hydraulic conductivity with two inputs (sand percentage and bulk density) during the model training and testing phases. The evaluation metrics R^2^, RMSE, and MBE yielded values of 0.556, 2.990, and 0.950 during the training phase and 0.392, 3.420, and 1.320 during the testing phase, respectively. In the model testing phase, the results were similar to those from the training phase. Here, the fuzzy model with inputs of percent sand, percent clay, percent silt, and bulk density had the highest correlation with the actual values, while the mean absolute error was also lower. Considering other evaluation criteria, the fuzzy model with inputs of percent sand and bulk density had the lowest correlation coefficient, indicating a relatively good fit with the measured values using fewer inputs. The ranking results of the models during the testing phase also indicated that the four-variable model with inputs of percent clay, percent sand, percent silt, and bulk density performed better (Table 3 and Figs. 2, 3, 4b). Moreover, the analysis also highlighted the critical influence of clay content on Ks estimation, while silt appeared to have a comparatively minor effect.

### ANN models

The ANN models, namely ANN_BFGS_ and ANN_C-G_, exhibited relatively poor performance in predicting saturated hydraulic conductivity (Table 3). During the training phase, the coefficient of determination (R^2^) for ANN_BFGS_ ranged from 0.156 to 0.297, while ANN_C-G_ ranged from 0.183 to 0.296, which are substantially lower than the DLLR model’s R^2^ values close to 0.99. This indicates a limited ability of these neural networks to accurately learn the underlying data patterns. Furthermore, the root mean square error (RMSE) during training was approximately between 0.42 and 0.45 for both models, considerably higher than the DLLR model’s RMSE of 0.004 to 0.0068. Mean bias error (MBE) values during training were close to zero, suggesting negligible systematic bias. In the testing phase, both ANN models showed no significant drop in performance compared to training; however, their overall accuracy remained low. The R^2^ values for ANN_BFGS_ ranged between 0.205 and 0.276, and for ANN_C-G_ between 0.125 and 0.293. The RMSE values during testing were between 0.41 and 0.43 for both models, indicating consistent prediction errors on unseen data. MBE values ranged between − 0.100 and − 0.062 for ANN_BFGS_, and − 0.0913 to 0.0247 for ANN_C-G_, reflecting a relatively balanced error distribution without significant over- or under estimation tendencies. These quantitative results clearly demonstrate that the current ANN models, under the present architectures and training parameters, lack sufficient capacity to generalize and accurately predict saturated hydraulic conductivity compared to the DLLR model. To enhance predictive performance, structural optimizations, parameter tuning, and potentially advanced data preprocessing techniques are necessary. Tian et al.^29^ reported significantly better predictive performance using multilayer perceptron (MLP) networks trained with backpropagation, achieving testing phase R^2^ values around 0.75 for saturated hydraulic conductivity estimation. This performance notably exceeds the relatively low testing R^2^ values (below 0.3) obtained in the present study for ANN_BFGS_ and ANN_C-G_ models. The discrepancy is likely attributed to larger and more diverse datasets and more optimized network configurations in their work.

### LR and DLLR models

The evaluation results of the regression models are presented in Table 4. Specifically, the R^2^, RMSE, and MBE values for the four-input regression model were 0.214, 3.488, and 0.0001 during the training phase and 0.301, 3.319, and 0.43 during the testing phase, respectively. The coefficients of the regression model are presented in Table 4. The results for the DLLR model, corresponding to R^2^, RMSE, and MBE, were 0.99, 0.0068, and 0.0037 during the training phase and 0.341, 0.428, and −0.159 during the testing phase, respectively.

**Table 4:** Linear regression model coefficients.

Model	Width from the origin	Model coefficients				R^2^_test_
		%Clay	%Silt	%Sand	P_b_	
1	− 13	0.100	0.231	0.251	− 2.933	0.301
2	− 3.4	–	0.128	0.147	− 2.933	0.302
3	0.83	–	–	0.058	− 0.021	0.189

1- Ks =−13—2.933 Bulk density + 0.251 sand% + 0.10 clay% + 0.231 silt%.

2- Ks =−3.40 + 0.128 silt% + 0.147 sand%−2.93 Bulk density.

3- Ks = 0.83 + 0.058 sand%−0.021 Bulk density.

The results of this study unequivocally indicate that the DLLR model significantly outperformed the linear regression (LR) model, achieving notably higher predictive accuracy and markedly lower error rates in estimating soil saturated hydraulic conductivity. This finding underscores the inherent limitations of simple linear approaches in representing the complex and nonlinear nature of soil hydraulic behavior. Moreover, the evaluation of model performance during the testing phase revealed that the DLLR model also surpassed the neural network models in predictive capability (Table 3). This superior performance of DLLR over neural networks aligns with the observations of Hekmatzad et al.^13^ and Merdun et al.^30^, further emphasizing the effectiveness of advanced regression techniques in modeling soil hydraulic properties. Collectively, these outcomes highlight that employing sophisticated nonlinear and local models such as DLLR constitutes a robust and reliable strategy for predicting soil hydraulic parameters, effectively addressing the shortcomings inherent in traditional linear modeling approaches.

### Comparative summary of models

**Fig. 5:**
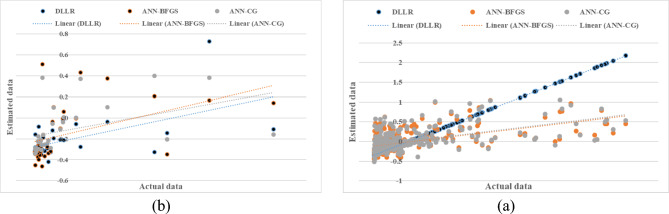
Comparison of the DLLR and ANN model’s calculated Ks with four inputs (**a**: model training) and (**b**: model testing).

**Fig. 6:**
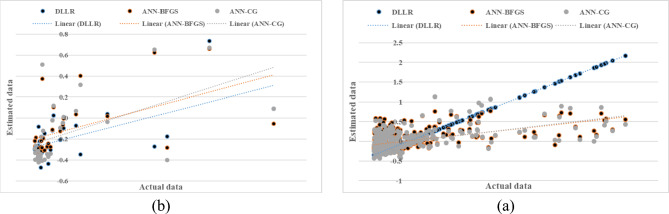
Comparison of the DLLR and ANN model’s calculated Ks with three inputs (**a**: model training) and (**b**: model testing).

**Fig. 7:**
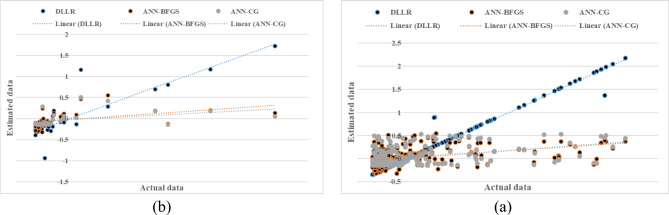
Comparison of the DLLR and ANN model’s calculated Ks with two inputs (**a**: model training) and (**b**: model testing).

The DLLR model exhibited superior performance in predicting saturated hydraulic conductivity, demonstrating both high accuracy and low error, as shown in Table 3 and Fig. 5, 6 and 7. A detailed comparison of the DLLR model with neural network models during the training and testing phases is presented in Figs. 5, 6, and 7. This superior performance aligns well with previous studies emphasizing the advantages of nonlinear and advanced modeling techniques. For example, Karahan and Erşahin^31^. and Tian et al.^29^ highlighted the effectiveness of fuzzy and machine learning-based approaches in capturing the complex behavior of soil hydraulic properties. Similarly, the exceptional performance of the DLLR model is consistent with findings from Durrant^20^, who advocated for the application of gamma tests and sophisticated nonlinear methods in hydraulic data analysis. Conversely, as noted by Pachepsky et al.^32^, linear regression models showed inferior performance due to their inability to adequately capture the inherent nonlinear relationships within soil data. Collectively, these findings corroborate the importance of employing advanced nonlinear models like fuzzy systems and DLLR to achieve more reliable and precise predictions of soil saturated hydraulic conductivity.

## Conclusion

Based on the presented results, it can be concluded that the developed fuzzy models have a relatively good fit with the measured values of saturated hydraulic conductivity, indicating their capability for estimation. According to the findings of this research, the observed discrepancies in estimating saturated hydraulic conductivity from the nonlinear model compared to actual values can be attributed to factors that, in addition to the parameters considered as model inputs, influence saturated hydraulic conductivity. However, since many factors affect the estimation of saturated hydraulic conductivity, selecting effective parameters and, if possible, eliminating some of them to reduce input variables can significantly aid in quick and cost-effective modeling. In this study, the results also showed a good correlation between the estimated saturated hydraulic conductivity from fuzzy models and the measured values, suggesting that this model can be used for estimating hydraulic conductivity.

Overall, this study highlights the importance of selecting advanced modeling approaches capable of capturing nonlinear and complex relationships in predicting saturated hydraulic conductivity. The DLLR and fuzzy models emerges as the most promising option, offering improved accuracy and reduced costs in soil hydrology and water engineering applications.

## Data Availability

The datasets used and analysed during the current study available from the corresponding author on reasonable request.
